# Cellular responses induced by multi-walled carbon nanotubes: *in vivo* and *in vitro* studies on the medicinal leech macrophages

**DOI:** 10.1038/s41598-017-09011-9

**Published:** 2017-08-21

**Authors:** Rossana Girardello, Nicolò Baranzini, Gianluca Tettamanti, Magda de Eguileor, Annalisa Grimaldi

**Affiliations:** University of Insubria, Department of Biotechnology and Life Sciences, Varese, 21100 Italy

## Abstract

The core characteristics of multi-wall carbon nanotubes (MWCNTs) are impressive and attractive for technology however, since their production and use is steadily increasing, their environmental dispersion could be potentially hazardous to animal and human health. For this reason, the identification of new methods and of reliable models to better understand MWCNT effects is essential. Here we propose the medicinal leech as an alternative model to assess the effects of MWCNTs on immune system. Our previous studies have already demonstrated that *in vivo* MWCNT treatment induces the activation of leech’s macrophages. Here we will focus on the direct effects of MWCNTs on these cells by isolating and culturing leech's macrophages by means of the consolidated Matrigel technique, followed by MWCNT *in vitro* treatment. Our results indicate that MWCNT administration causes both the decrease of cell proliferation rate and the increase of the apoptotic rate. Furthermore, since oxidative stress is linked with inflammation, reactive oxygen species has been evaluated confirming that their production rate increases after MWCNT treatment. Our experimental approaches demonstrate the ability of MWCNTs inducing a powerful inflammatory response and confirm that the medicinal leech is a good alternative model to study the possible harmful effects of any nanomaterial.

## Introduction

Multi-walled carbon nanotubes (MWCNTs), consisting of several concentric graphene tubes, with diameters of up to 100 nm, are one of the most promising classes of nanomaterials today and are used for many industrial developments, nanoelectronics, mechanical engineering and biomedical applications^1–3^. Given their high production and large use, the environmental exposure to this nanomaterial is supposed to increase dramatically in the next years as well as their release in both terrestrial and aquatic environment^4–6^. The potential harmful impacts of MWCNTs are indeed attracting increasing research and public attention^7, 8^. It needs to be remarked that the essential characteristics of MWCNTs, i.e. the high biological stability and not degradability in biological systems for months or even years^9–12^, may pose hazards similar to those of other fine or ultrafine particles and fibers, such as diesel exhaust particles and asbestos^10^ as already observed in the past. Indeed a certain amount of *in vitro* and *in vivo* studies demonstrates MWCNT toxicity and biopersistence within cells and tissues^13–15^. Moreover several researches provided evidences in support of MWCNT pro-inflammatory, pro-fibrotic, pro-angiogenic^16–18^, cytotoxic and oxidative stress effects^19^, leading to a dose and time-dependent production of reactive oxygen species (ROS) and a reduction in cell viability and even inducing apoptosis^20–22^.

To recap the above: all of the data in literature point out that both organism’s immune system and ROS production could represent sensitive physiological indicators affected by MWCNT exposure. Since they give information on animal physiological status, they can indirectly provide information on environmental health and pollution. Therefore, they could be used as biomarkers to simply and quickly evaluate the effects induced by MWCNT exposure.

This study suggest the use of the medicinal leech *Hirudo verbana*, a species closely related to the best-known *Hirudo medicinalis*, as a new aquatic invertebrate model to develop a simple, common and quick method to detect MWCNT dispersion in an aquatic environment and to evaluate their effects measuring ROS production and cell viability. It is well known that invertebrates orchestrate rapid and sensitive responses upon the presence of pollution. Our previous works^17, 18^ have demonstrated that leech activates a rapid immune response in presence of MWCNTs. As already largely described, this model presents multiple advantages, being cost-effective, easily manipulable and devoid of significant ethical considerations and regulatory restrictions in relation to its use^23, 24^. Moreover, the immune response processes in leeches have proven to be surprisingly similar to those reported in Vertebrates^25–27^ more than in most commonly used invertebrate models such as *Drosophila melanogaster* or *Caenorhabditis elegans*. Its anatomical and physiological characteristics and a less varied repertoire of cell types involved in immune response allow to easily interpreting and unambiguously evaluating events linked to immune response evoked by different stimuli^26, 28–31^.

## Results

### Cell proliferation assay

**Figure 1:**
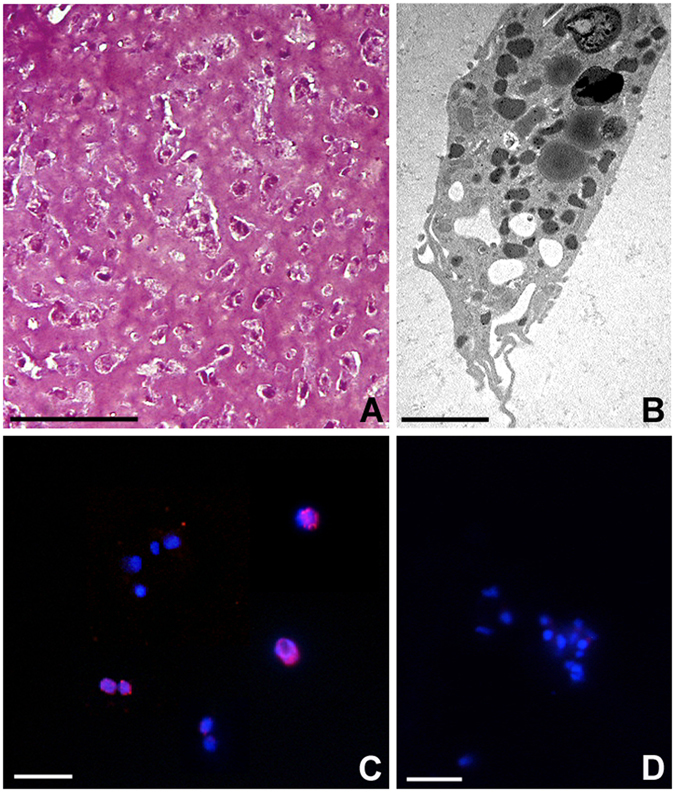
Phenotype analysis of cells recruited into the matrigel sponges by *Hm*AIF-1 (**A**,**B**) and proliferation assay (**C**,**D**). After 1 week *in vivo*, MG is infiltrated by numerous cells stained by crystal violet and basic fuchsine (**A**). Ultrastructural analysis at TEM (**B**) confirm the macrophagic nature of the migrating cells in the MG sponges. Immunofluorescence experiment (**C**) shows BrdU^+^ nuclei (in red) in most of the cell migrated in the MG sponges. No signal is detected in negative control experiments (**D**) where the primary antibody was omitted. Nuclei are counterstained with DAPI (blue). Bar in (**A**) 50 µm; bar in (**B**) 2 µm; bars in (**C**,**D**) 10 µm.

In order to assess the effects of MWCNTs on leech macrophage proliferative activity, macrophages were detected and isolated from leech body wall by means of the biopolymer matrigel (MG) supplemented with the recombinant protein r*Hm*AIF-1^18^, a specific macrophage chemoattractant homolog to human allograft inflammatory factor-1 (AIF-1), recently identified and characterized in the medicinal leech^30^. At first MG solid pellets, formed following inoculation and containing *Hm*AIF-1 protein, were explanted after 1 week and then processed for standard histological analysis. MG samples appeared massively infiltrated, at optical microscope, by numerous cells with an irregular shape and an active degradation of the surrounding matrix (Fig. 1A). Ultrastructural analysis at transmission electron microscope (TEM) showed that the infiltrating cells were macrophages characterized by a cytoplasm filled with phagolysosomes and highly electron-dense granules. The migratory phenotype of these cells was confirmed by the presence of pseudopodia (Fig. 1B). In order to assess whether, once trapped in MG, the cells infiltrating the matrigel retained their proliferative ability, the 5-Bromo-2′-deoxy-uridine (BrdU) assay was foremost performed. Immunofluorescence experiments showed that the nuclei of the most part of macrophages migrated to the MG sponge were BrdU^+^ (Fig. 1C). In the negative control experiments, when the primary antibody was omitted, no BrdU signal was observed (Fig. 1D).

In order to evaluate the effects of different concentration of MWCNTs on cultured leech macrophage cells, the MG polymers supplemented with r*Hm*AIF-1 were removed from the body wall of leeches after 1 week *in vivo* and the cells were placed in culture.

**Figure 2:**
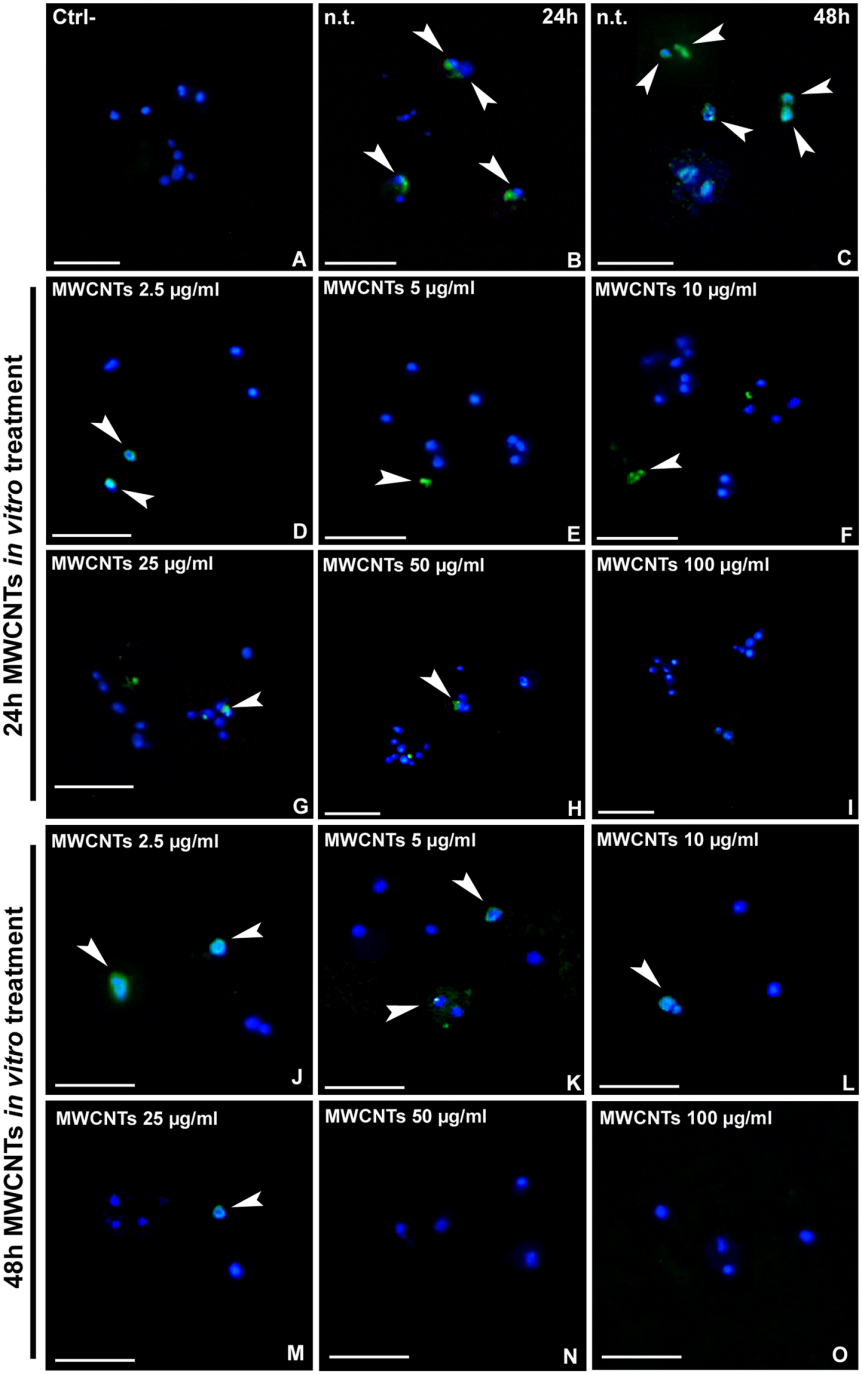
Proliferation assay after MWCNT *in vitro* treatment (**A**–**O**). Compared to the negative control (**A**), where the primary antibody was omitted, the major part of cells in n.t. samples are BrdU^+^ (arrowheads) both at 24 h (**B**) and 48 h (**C**). In MWCNT treated samples, the number of positive cells decreases in dose and time dependent way (**D**–**O**). Nuclei are stained with DAPI (blue). Bars in (**A**–**O**) 20 µm.

After obtaining primary culture of macrophages, cells were exposed to different MWCNT concentrations (Fig. 2A–O). 24 hours after seeding, a large number of BrdU^+^ cells was observed in control untreated samples (Fig. 2B). This percentage remained high even after 48 hours from seeding (Fig. 2C). On the other hand the number of cultured proliferating cells became highly reduced after 24 (Fig. 2D–I) and 48 hours (J-O) from MWCNT administration. In particular, a high dose of MWCNTs (100 µg/mL) totally inhibited cell proliferation and no BrdU^+^ cell was detectable after treatment (Fig. 2I–O). During the control experiments, when the primary antibody was omitted, no BrdU^+^ cells were detectable (Fig. 2A).

**Figure 3:**
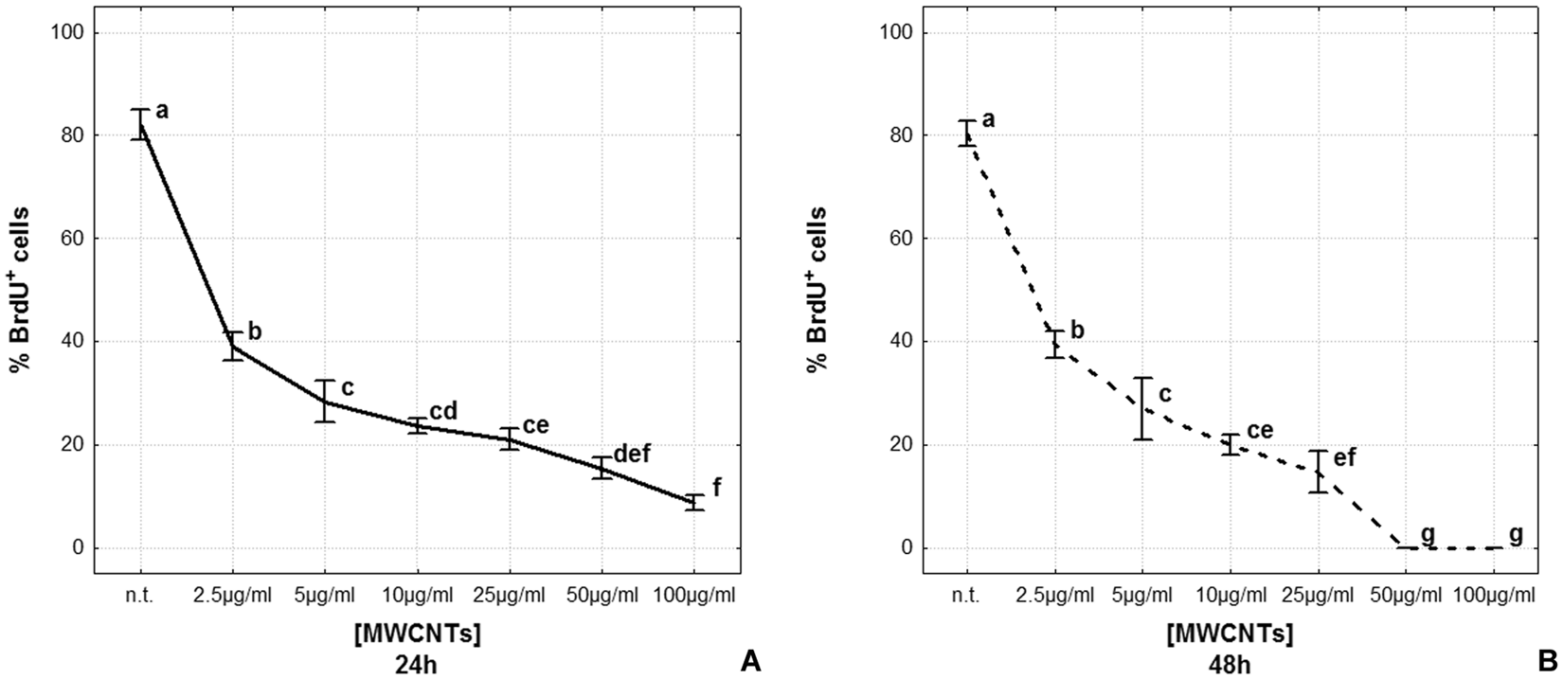
Cell proliferation rate after MWCNT *in vitro* treatment (**A**,**B**). The graphs show the percentage of BrdU^+^ cells after 24 h (**A**) and 48 h MWCNT treatment (**B**). Statistical differences were calculated by Factorial ANOVA followed by Tukey’s post-hoc test; vertical bars denote standard deviation, different letters indicate statistically significant differences (p < 0.05).

Cell counting (Fig. 3A,B) confirmed a significant decrease in BrdU^+^ cells even at low doses (2.5, 5 and 10 µg/ml) of MWCNTs at both 24 and 48 hours. Indeed the effect is even more evident after 48 hours from MWCNT administration at higher doses (25, 50 and µg/mL). (% BrdU^+^ cells is represented as mean ± s.d.; 24 hours samples: n.t.: 81.94 ± 3.00; 2.5 µg/ml: 39.00 ± 2.65; 5 µg/ml: 28.19 ± 4.01; 10 µg/ml: 23.74 ± 1.41; 25 µg/ml: 21.03 ± 2.04; 50 µg/ml: 15.44 ± 1.90; 100 µg/ml: 5.71 ± 1.59 [p < 0.00001]; n = 21, Fig. 3A. 48 hours samples: n.t.: 80.33 ± 2.52; 2.5 µg/ml: 39.33 ± 2.62; 5 µg/ml: 27.11 ± 6.17; 10 µg/ml: 20.07 ± 2.11; 25 µg/ml: 14.76 ± 4.02; 50 µg/ml: 0.00 ± 0.00; 100 µg/ml: 0.00 ± 0.00 [p < 0.00001]; n = 21, Fig. 3B).

### Apoptosis detection assay

**Figure 4:**
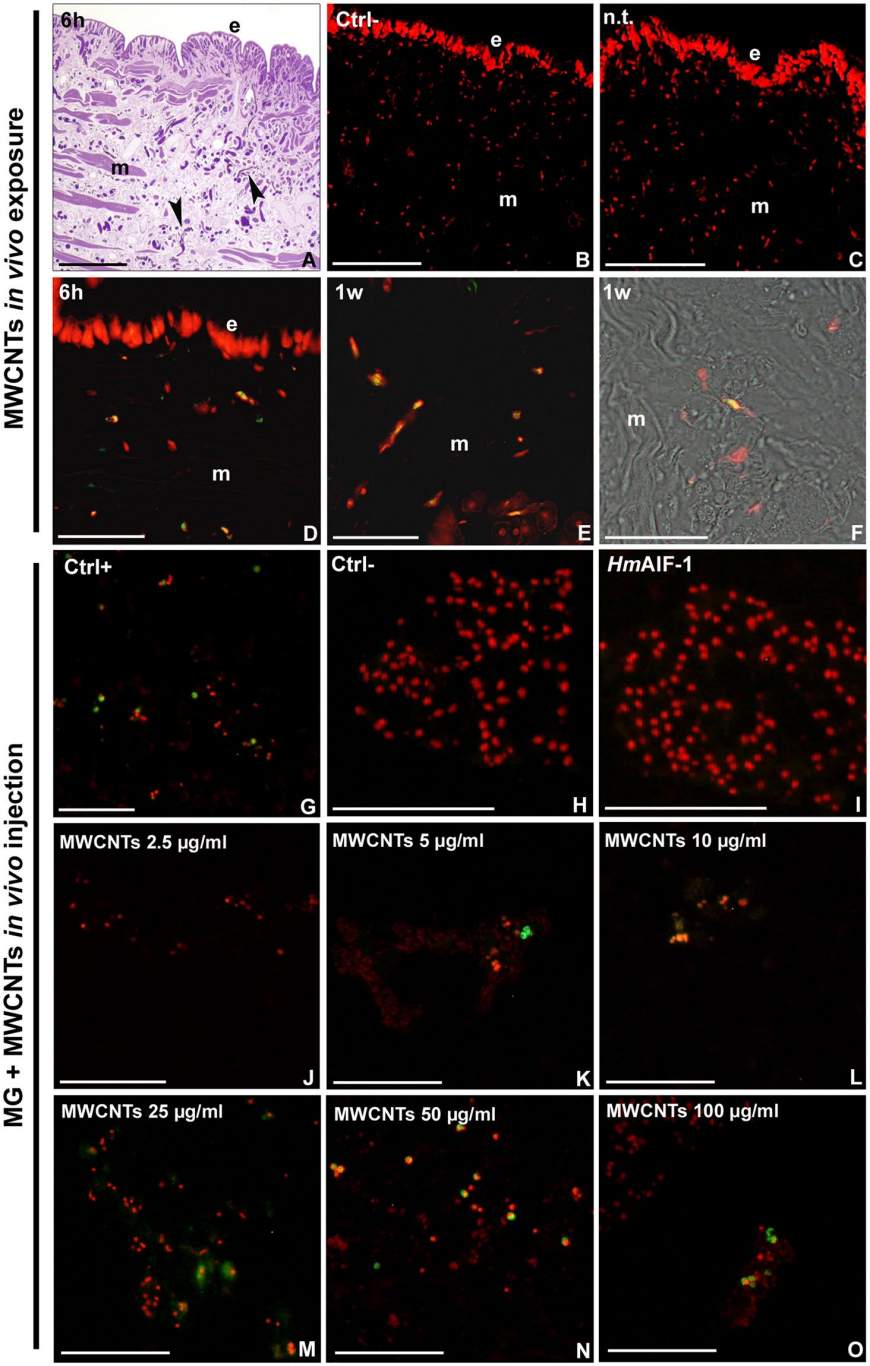
TUNEL assay for apoptotic nuclei detection in leech body wall (**A**–**F**) and in MG sections (**G**–**O**). Optical images showing numerous macrophages (arrowheads) migrating in the leech body wall after MWCNT treatment (**A**). Negative control (**B**). N.t. (**C**) and MWCNT treated leeches (**C**–**E**). Combined optical and fluorescence images of leech body wall showing TUNEL^+^ cells migrating among muscle fibers (**F**) (e: epithelium; m: muscles). Positive (**G**) and negative controls (**H**) on MG samples. MG supplemented with *Hm*AIF-1 (**I**) and with *Hm*AIF-1 and increasing concentration of MWCNTs (**J**–**O**). Nuclei, counterstained with propidium iodide result in red while TUNEL positivity in green. The merge results in yellow. Bars in (**A**–**C**,**G**–**O**) 100 µm; Bars in (**D** **–** **F**) 50 µm.

As already demonstrated in our previous work, MWCNT treatment induced a massive migration of macrophages in the leech body wall, indicating a strong inflammatory response^17^. These cells were mainly localized underneath the epithelium and among fields of muscle fibers (Fig. 4A). Terminal deoxynucleotidyl transferase dUTP nick-end labeling (TUNEL) assay was performed on tissues of both untreated (Fig. 4B,C) and MWCNT exposed leeches (Fig. 4D–F). In the negative control (Fig. 4B) experiment, performed as described in Material and Methods section, as well as in n.t. leech tissues (Fig. 4C), no apoptotic nuclei were detected. Otherwise, TUNEL^+^ nuclei were visible after 6 hours (Fig. 4D) and 1 week (Fig. 4E) from MWCNT exposure. The apoptotic cells were mainly localized among muscle fibres and underneath the epithelium (Fig. 4F).

To confirm the direct involvement of MWCNT cell treatment in inducing apoptosis, a MG assay was also performed. MG supplemented only with *Hm*AIF-1 or MG supplemented with both MWCNTs and *Hm*AIF-1 were injected in the leech body wall. The MG pellets that were formed following inoculation were recovered and processed for TUNEL test 1 week after injection. Results showed that TUNEL^+^ (green/yellow) nuclei were visible in the positive control sample (Fig. 4G), while in the negative control no signal was detected (Fig. 4H). No evidence of apoptosis was detected in the cells migrated in MG supplemented with *Hm*AIF-1 alone (Fig. 4I), whereas in MWCNT supplemented MG samples the number of TUNEL^+^ nuclei increased in a dose-dependent manner (Fig. 4J–O).

**Figure 5:**
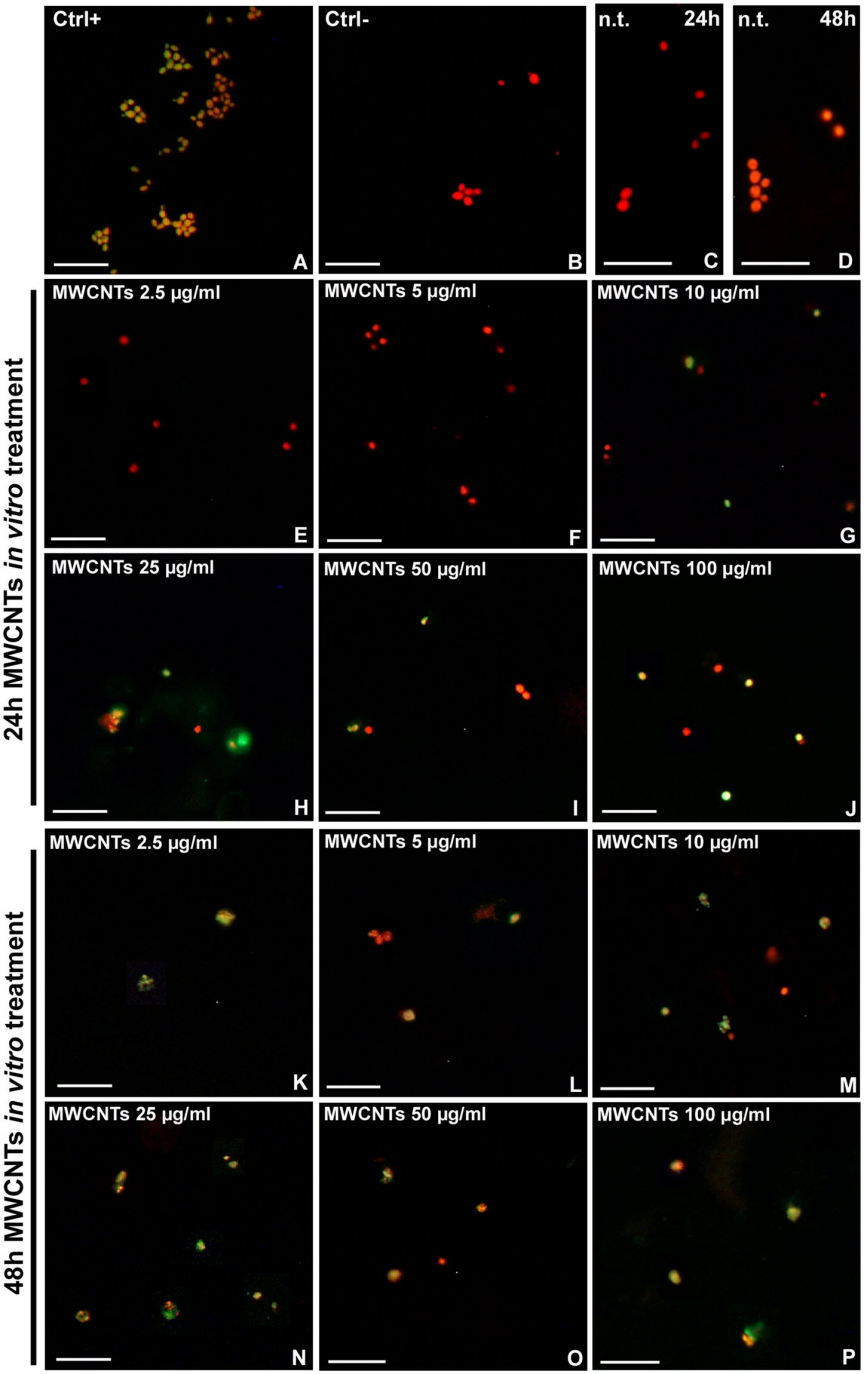
TUNEL assay for apoptotic nuclei detection after *in vitro* MWCNT treatment (**A**–**P**). DNase treated positive control (**A**). Negative control in which the primary antibody was omitted (**B**) and 24 h and 48 h n.t. samples (**C**,**D**). Cultured macrophages 24 h (**E**–**J**) and 48 h (**K**–**P**) after MWCNT *in vitro* treatment. Nuclei were counterstained with propidium iodide (red). TUNEL positivity is visible in green/yellow. Bars in (**A**–**P**) 20 µm.

The apoptosis evaluation assay was also performed on primary culture of macrophages, obtained as described above. Apoptotic nuclei were highlighted in yellow, as confirmed by the positive control (Fig. 5A), while all nuclei were counterstained in red by propidium iodide, as visible in the negative control (Fig. 5B). In n.t. samples (Fig. 5C,D) as well as after 24 hours from treatment with 2.5 and 5 µg/ml of MWCNTs (Fig. 5E,F), no apoptotic nuclei were visible. The number of TUNEL^+^ cells increased remarkably after administration of higher doses of MWCNTs (Fig. 5G–J). After 48 hours from MWCNT administration (Fig. 5K–P), apoptosis was induced in a large number of cells at any concentrations.

**Figure 6:**
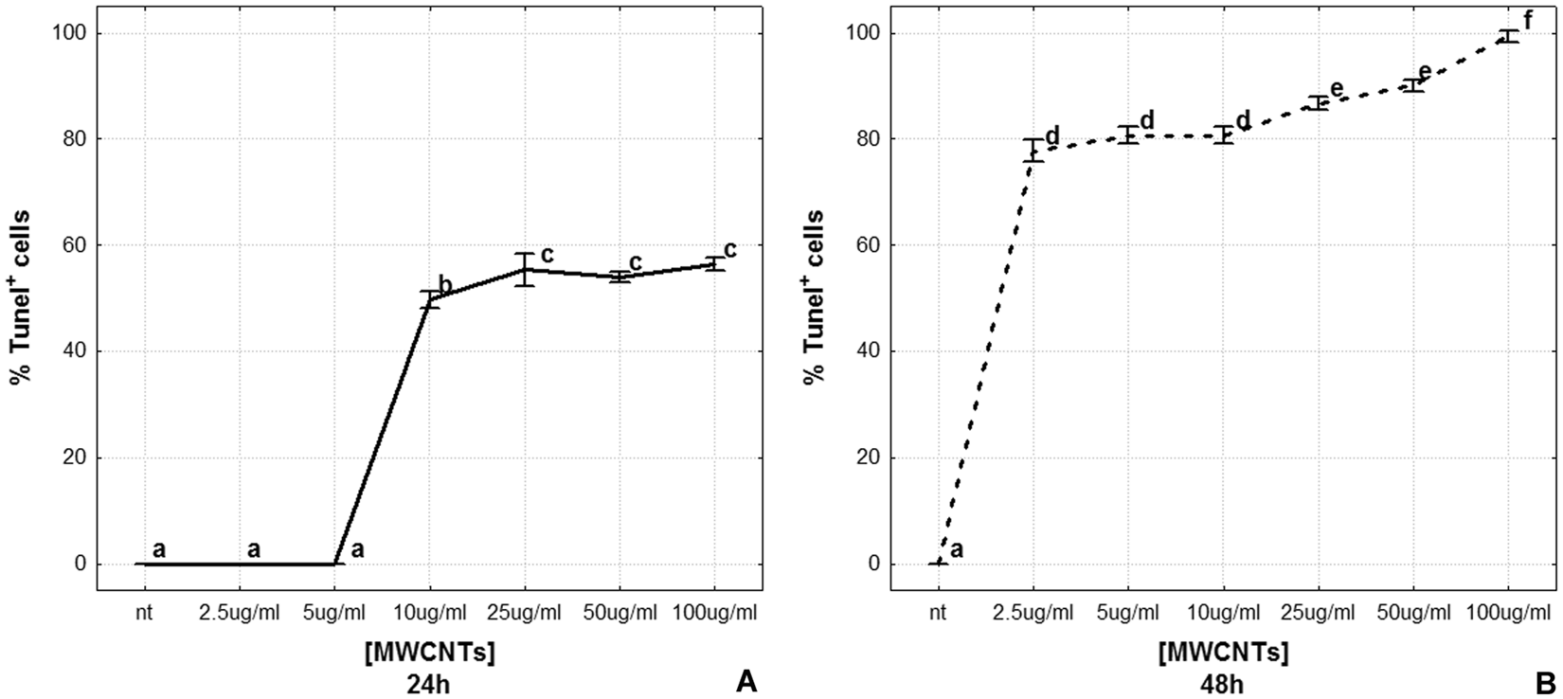
Apoptotic rate after MWCNT *in vitro* treatment (**A**,**B**). The graphs illustrate the percentage of TUNEL^+^ cells 24 h (**A**) and 48 h (**B**) after *in vitro* MWCNT treatment. Statistical differences were calculated by Factorial ANOVA followed by Tukey’s post-hoc test; vertical bars denote standard deviation, different letters indicate statistically significant differences (p < 0.05).

Cell counting showed that apoptotic cell number was very low in n.t. and low-dose MWCNT treated samples (2.5 and 5 µg/ml) for 24 hours. At the dose of 10 µg/ml, it underwent a sudden increase to then reach about 60% of total cells at higher doses (% Tunel^+^ cells is represented as mean ± s.d.; n.t.: 0.00 ± 0.00; 2.5 µg/ml: 0.00 ± 0.00; 5 µg/ml: 0.00 ± 0.00; 10 µg/ml: 49.67 ± 1.53; 25 µg/ml: 55.33 ± 3.06; 50 µg/ml: 54.00 ± 1.00; 100 µg/ml: 56.33 ± 1.15 [p < 0.00001]; n = 21, Fig. 6A).

The 48 hours MWCNT treatment seemed to have a more rapid effect. TUNEL^+^ cells were about the 80% at the lowest dose (2.5 µg/ml) and reached the 100% with the 100 µg/ml treatment (% Tunel^+^ cells is represented as mean ± s.d.; 48 hours samples: n.t.: 0.00 ± 0.00; 2.5 µg/ml: 77.80 ± 1.92; 5 µg/ml: 80.57 ± 1.47; 10 µg/ml: 80.84 ± 1.37; 25 µg/ml: 86.78 ± 0.96; 50 µg/ml: 90.02 ± 1.14; 100 µg/ml: 99.17 ± 1.44 [p < 0.00001]; n = 21, Fig. 6B).

### ROS production

**Figure 7:**
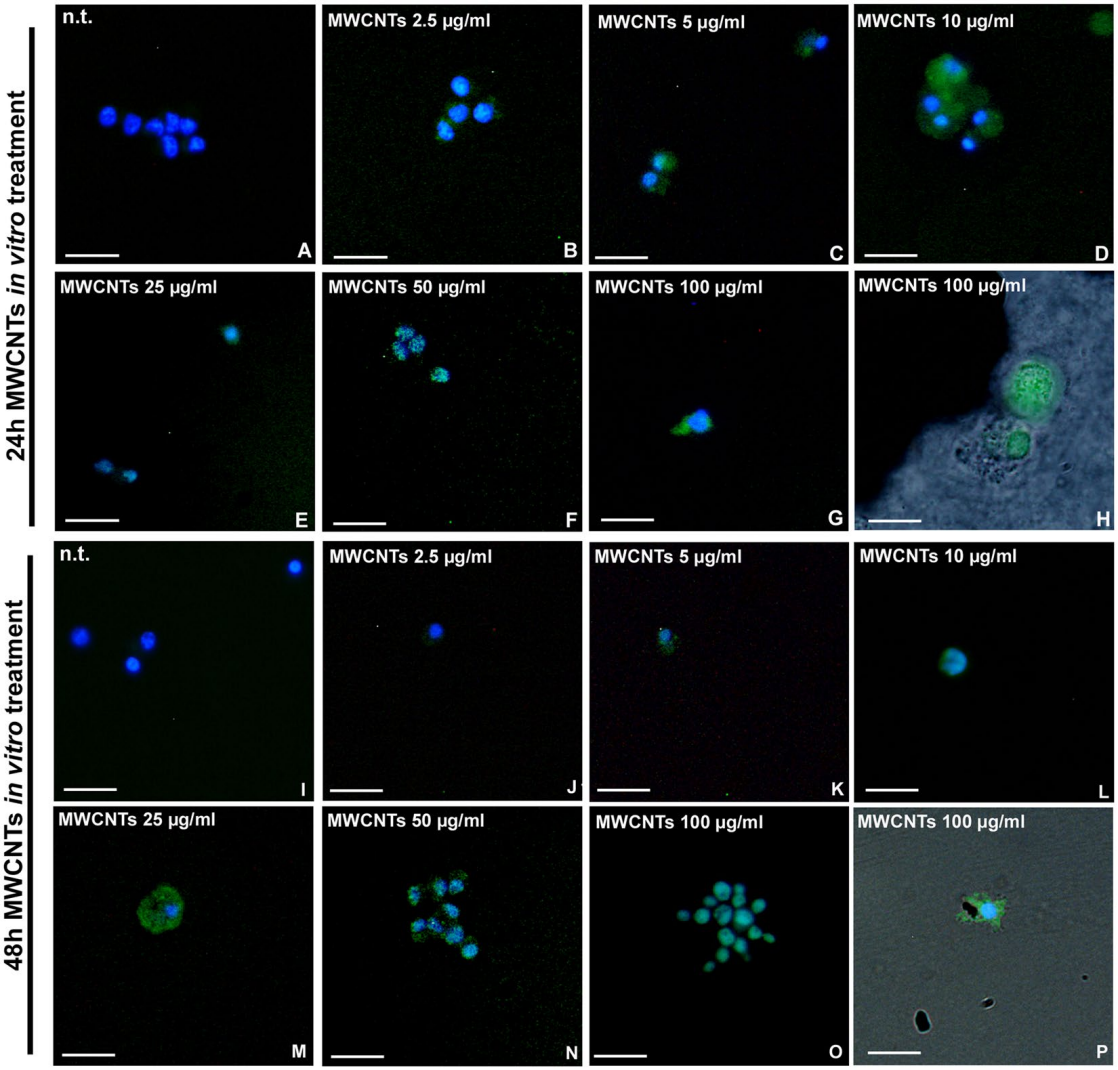
H_2_DCFH-DA assay for ROS detection (**A**–**P**). H_2_DCFH-DA positivity (green) is not visible in n.t. macrophages at 24 h (**A**). After 24 h *in vitro* treatment with increasing concentration of MWCNTs a strong positivity is detectable (**C**–**H**). The same dose-dependent ROS production is appreciable after 48 h from treatment (**J**–**P**). Combined optical and fluorescence details showing H2DCFDA^+^ cells close to MWCNT bundles (**H**,**P**). Bars in (**A**–**P**) 10 µm.

ROS production was evaluated *in vitro* by means of 2′,7′-dichlorodihydrofluorescein diacetate (H_2_DCFDA) assay. In n.t. cells after 24 hours (Fig. 7A) no signal was detected. After 24 hours MWCNT *in vitro* treatment, ROS production was observed starting from concentrations of 2.5 µg/ml up to 100 µg/ml (Fig. 7B–G). After 48 hours, n.t. cells showed no sign of ROS production (Fig. 7I) while a high H_2_DCFDA positivity was observed in MWCNT treated cells (Fig. 7J–O). In particular, ROS signal was localized in cells in close contact with MWCNT bundles (Fig. 7H,P).

**Figure 8:**
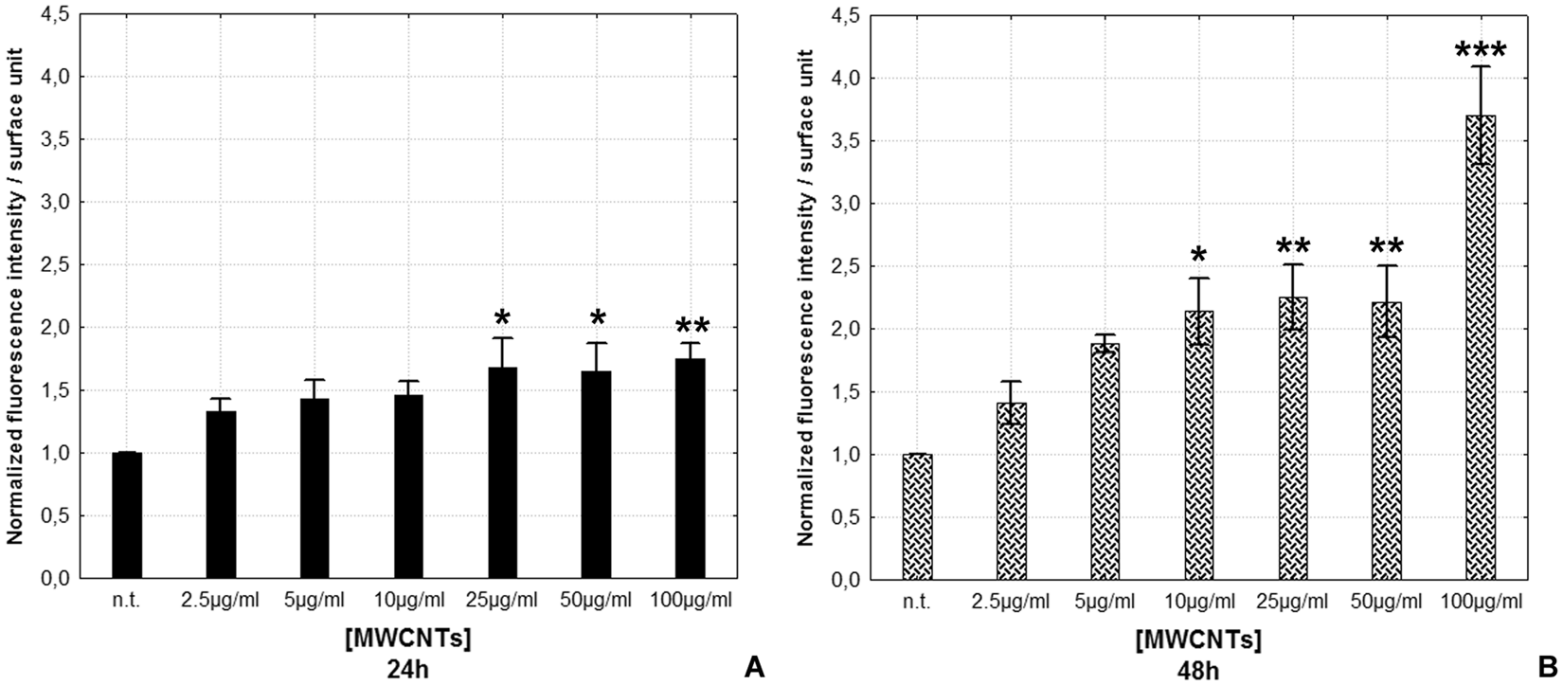
ROS production evaluation (**A**,**B**). Histograms showing H_2_DCFH-DA assay fluorescence intensity for surface unit after 24 h (**A**) and 48 h (**B**) MWCNT treatment. Statistical differences were calculated by Factorial ANOVA followed by Tukey’s post-hoc test, vertical bars denote standard deviation, *p < 0.05, **p < 0.01, ***p < 0.001 (between n.t. and treatments).

Fluorescence intensity measurement showed a significant increase in ROS production after 24 hours from treatment with high concentrations (25, 50 and 100 µg/ml) of MWCNTs (Fluorescence intensity for surface unit after H_2_DCFHDA assay is represented as mean ± s.d.; 24 hours samples: n.t.: 1.00 ± 0.00; 2.5 µg/ml: 1.15 ± 0.08; 5 µg/ml: 1.15 ± 0.10; 10 µg/ml: 1.15 ± 0.14; 25 µg/ml: 1.43 ± 0.33; 50 µg/ml: 1.60 ± 0.39; 100 µg/ml: 1.40 ± 0.10 [p = 0.0180]; n = 21, Fig. 8A), while after 48 hours, the increase of ROS was already evident starting from the 10 µg/ml dose up to the highest dose (48 hours samples: n.t.: 1.00 ± 0.00; 2.5 µg/ml: 1.41 ± 0.17; 5 µg/ml: 1.88 ± 0.07; 10 µg/ml: 2.08 ± 0.34; 25 µg/ml: 2.43 ± 0.31; 50 µg/ml: 2.81 ± 0.34; 100 µg/ml: 3.64 ± 0.47 [p = 0.0002]; n = 21, Fig. 8B).

## Discussion

As previously demonstrated^17, 18^, *Hirudo* results a good model to study MWCNT effects, because of its very rapid and clearly detectable immune response. Our recent studies show that the MWCNT environmental exposure causes in the leech an extremely rapid inflammatory response suggesting the ability of this NM to overcome superficial barriers. Indeed, after MWCNT treatment, high levels of the pro-inflammatory cytokine Interleukin 18 (IL-18) are expressed by numerous macrophage-like cells, typically expressing different cluster of differentiation antigens, such as CD68 and CD45, and *Hm*AIF-1^17^. These cells, once activated, migrate towards the stimulated area and produce amyloid deposits in correspondence of MWCNT aggregates. As already demonstrated in our previous paper, amyloid fibrils are easy detectable by using the colorimetric methods of Thioflavin-T^17, 18^. It need to be stressed that the production of amyloid fibrils, normally associated with neurodegenerative diseases, is necessary in this case for the formation of a scaffold around MWCNT aggregates, to create a barrier to contain the exogenous material^32^.

Since Atomic Absorption Spectroscopy and Scanning electron microscopy coupled with X-ray spectroscopy analyses confirm that metal impurities associated to MWCNTs are neither released after resuspension, nor detected in leech tissue samples after exposure, our data suggest that the immune reactions observed in MWCNT exposed leeches are caused specifically by MWCNTs and not by associated impurities^16, 17^.

Starting from these results mainly obtained from *in vivo* experiments, *in vitro* studies become necessary to better clarify the interactions between MWCNTs and macrophages and the effects of this NM on immune cells. In particular, since previous ultrastructural analysis demonstrate that MWCNTs are be able to pierce cell membranes, we hypothesis a possible effect of MWCNTs in inducing an increase of intracellular ROS levels and consequently to the onset of cell death mechanisms^18^. In this study, we focused on these aspects, and in particular, we investigated the direct effect on macrophage proliferation, apoptosis and ROS production induced by MWCNT exposure by means of simple and fast assays, such as H_2_DCFDA staining, to evaluate ROS production, BrdU assay and TUNEL test to assess cell proliferation and apoptotic rates respectively.

The proliferation assay on MG sponges demonstrates that our consolidated experimental approach for macrophage isolation^18, 33, 34^ does not cause damages to cells, which retain their proliferative ability. On the other hand, after MWCNT *in vitro* treatment this ability significantly decreases.

Given that at our knowledge, no data attesting the real concentrations of MWCNTs present in the environment are actually present in literature^4, 7, 35^, we designed *in vitro* studies using different concentrations of MWCNTs. Since until now are not available the MWCNT environmental concentration, to better elucidate mechanisms/mode of actions suggested by studies in whole animals, we tested *in vitro* a range of concentrations starting from a low dose (i. e. 2.5 μg/ml) up to the worst-case scenario (i. e. 100 μg/ml). As revealed by TUNEL assay, we show that both MWCNT environmental dispersion and injection are able to induce apoptosis. Moreover, our *in vitro* approach lead us to confirm that there is a correlation between apoptosis and MWCNT treatment, and that the observed effect is dose and time dependent. These data are consistent with other studies indicating the induction of apoptosis as an indicator of NM toxicity^36^. In addition, we provide evidences of an increase in ROS production after MWCNT exposure. Indeed ROS increase is generally considered a major contributor to carbon nanotubes toxicity^13, 37^, and we assume that the high level of measured ROS at high concentration may have been the result of cell death. In particular, the trend observed for ROS production after MWCNT exposure is strictly linked to the fact that, after treatment with MWCNT lower concentrations (from 10 up to 50 μg/ml), not all cells are in oxidative stress. However, after treatment with 100 μg/ml of MWCNTs, ROS production dramatically increases in concomitance with the peak in number of apoptotic cells. Indeed it has been previously reported that ROS is essential for numerous physiological control of cell function, and it is required for cell cycle entry and proliferation^38–40^. Given that an excessive oxidative stress is harmful to cells, normal cells have an antioxidant capacity for appropriate redox state retention to control it. Thus, it is expected that in cells treated with lower concentrations and shorten exposure time of MWCNTs, intracellular ROS level increased, but thanks to the action of a ROS scavenger system, cells were able to maintain a constant, although higher than the control cells, concentration of intracellular ROS level. However, the elevated ROS production in higher concentrations and longer exposure time treated cells could be due to severe DNA damage induced by MWCNT treatment and related to the redox system and DNA repair system failure and apoptosis mechanism activation^41^.

Moreover, since MWCNTs have been observed in the cytoplasm of cells showing nor morphological alterations nor membrane ruptures, we are confident that ROS and apoptosis are induced by MWCNT entrance and not by membrane damages.

In conclusion, our study provides evidences to support that MWCNTs are able to induce, both *in vivo* and *in vitro*, a plethora of inflammatory responses leading cells to a strong production of ROS, a reduced proliferation rate and the onset of programmed cell death pathways. In particular, *in vitro* treatment demonstrated that the observed responses are correlated to both MWCNTs doses and exposure time. Taken together, our results represent another relevant piece in the puzzle of nanomaterial toxicity.

Finally, it is important to emphasize that nowadays the scientific community has the urgent need to reduce *in vivo* study in accordance with international guidelines concerning animal testing^22^. However, many of the *in vitro* studies, using cancer cell lines, do not take into account the typical intrinsic resistance to apoptosis of cancer cells^36^. Our original and innovative assay based on the use of the biomatrix matrigel (MG) to obtain *in vitro* expansion of leech macrophages^18, 33, 34, 42^, which are also implicated in response against abiotic particles such as MWCNTs, is an important tool that can be used as quick sensitive model for aquatic pollution bio-monitoring. Thus, in living organisms and culture cells, measurement of ROS intracellular levels and cell proliferation and viability might provide a reliable method to quickly identify oxidative stress states induced by the presence of MWCNTs in the environment.

Given the above facts, our data represent a cornerstone in the determination of the toxicity of pristine MWCNTs and furthermore demonstrate the relevance of an alternative model to study both *in vivo* and *in vitro*, the possible harmful effects of any nanomaterial.

Moreover, since autophagic cell death pathway activation is emerging as a possible consequence of MWCNT treatment^43^, we will try to clarify this aspect to understand completely the MWCNT- induced toxicity.

## Materials and Methods

### MWCNT characteristics and preparation

NANOCYL™ NC7000 carbon nanotubes were obtained from NANOCYL (Belgium, Sambreville). Their dimensions is equivalent to 9.5 nm external diameter by 1.5 μm mean length with a specific surface area ranging from 250 to 300 m^2^/g. NC7000 were manufactured by a CCVD (Catalytic Carbon Vapor Deposition) process. No chemical modification was applied to the MWCNTs before use. Pristine MWCNTs powder was weighed, suspended in water or culture medium, depending on the subsequent experimental plan, and then sonicated in an ultrasonic bath 15 min for two cycles (28–34 KHz) to avoid aggregation of particles. No surfactant was used for dispersing MWCNTs.

MWCNT crude powder and MWCNT suspensions in water or cell culture medium (RPMI-1640 (EuroClone SpA, Milan, Italy) supplemented with 1% glutamine, 10% fetal bovine serum, 1% penicillin-streptomycin and 1% gentamicin) were characterized by transmission electron microscopy (TEM), scanning electron microscopy (SEM), energy dispersive X-ray spectroscopy (EDS) and Atomic Absorption Spectroscopy (AAS) analyses, as per our previous paper^17^.

MWCNT concentrations and exposure time were determined based on previous data in literature^44, 45^ according to the exposure type.

### *In vivo* assay

Adult leeches (*H*. *verbana*, Annelida, Hirudinea, from Ricarimpex, Eysines, France), were kept in water at 20 °C in aerated tanks and exposed to MWCNTs (400 mg/L). Controlled not treated (n.t.) animals were kept without MWCNTs. Controlled and MWCNT exposed leeches at specific time points (6 hours and 1 week) were anesthetized with 10% ethanol solution and sacrificed. Leeches were then dissected and body wall tissues, at 20th metamere level, were fixed with paraformaldehyde 4% for 1 h at room temperature. After standard ethanol dehydration, samples were embedded in paraffin (Bioptica, Milan, Italy) and then cut with a Leica Jung Multicut 2045 Microtome (Leica, Nussloch, Germany).

### Matrigel assay

Leeches were injected with 300 µl of MG supplemented with 300 ng of r*Hm*Aif-1 and 2.5, 5, 10, 25, 50 and 100 µg/ml of Multi-walled Carbon Nanotubes (MWCNTs), NANOCYL^TM^ NC7000 (Belgium NANOCYL, Sambreville; average 9.5 µm diameter by 1.5 µm, not functionalized, manufactured by Catalytic Carbon Vapor Deposition (CCVD) process, with a purity of 90%). Leeches injected with 300 µl of MG supplemented only with r*Hm*Aif-1 were used as a not treated control (n.t.). Before the injection, performed at 20th metamere level, leeches were anaesthetized with a 10% ethanol solution. MG implants were removed from the animal after 1 week and processed in different ways depending on the type of assay in which they were intended.

### *In vitro* assay

After 1 week *in vivo*, MG implants were harvested and cultured. Each implant was plated in wells of 60 mm in diameter in RPMI-1640 medium (EuroClone SpA, Milan, Italy) supplemented with 1% glutamine, 10% fetal bovine serum, 1% penicillin-streptomycin and 1% gentamicin. Cells were maintained at 20 °C for 1 week before MWCNT treatment. MWCNTs were re-suspended in complete culture medium at 2.5, 5, 10, 25, 50 and 100 μg/ml. To avoid particles aggregation, the solutions were sonicated 15 min for 2 cycles in an ultrasonic bath (starsonic 35, Liarre, Italy) immediately before administration. Cells were treated for 24 and 48 hours and then processed in different ways depending on the type of analyses.

### Optical and Trasmission Electron Microscopy (TEM)

Tissues from leech body wall and MG pellets were fixed for 2 h in 0.1 M cacodylate buffer at pH 7.4, containing 2% glutaraldehyde. After three washes in the same buffer, specimens were post-fixed for 1 hour with 1% osmium tetroxide in cacodylate buffer, pH 7.4. After standard ethanol dehydration, all samples were embedded in an Epon-Araldite 812 mixture. Sections were obtained with a Reichert Ultracut S ultratome (Leica, Wien, Austria). Semi-thin sections (0.75 μm in thickness), stained by crystal violet and basic fuchsine^46^ were observed under the light microscope Nikon Eclipse Ni (Nikon, Tokyo, Japan). Data were recorded with a DS-5M-L1 digital camera system Nikon. Ultrathin sections (80 nm in thickness) were stained by uranyl acetate and lead citrate and observed with a Jeol 1010 EX electron microscope while data were recorded with a MORADA digital camera system (Olympus, Tokyo, Japan).

### Terminal deoxynucleotidyl transferase dUTP nick-end labelling (TUNEL) assay

The DeadEnd™ Fluorometric TUNEL System (Promega, Pittsburgh, PA, USA) was used to evaluate the presence of apoptotic cells in the different samples. Leech tissues sections were deparaffinised, rehydrated and then permeabilized with a 20 μg/ml Proteinase K solution in PBS for 10 minutes at room temperature.

MG cryosections and *in* *vitro*-treated macrophages were fixed in paraformaldehyde 4% for 25 minutes at 4 °C and then washed in PBS and permeabilized in 0.2% Triton X-100 in PBS for 5 minutes. According to the manufacturer’s protocol, after an equilibration step, we added the incubation buffer containing nucleotide mix and rTdT enzyme. Slides were incubated at 37 °C for 1 hour in the dark and then immersed in a 1x sodium chloride and Sodium Citrate buffer (SSC). Nuclei were counterstained with 1 µg/ml Propidium Iodide (Sigma Aldrich, Saint Luis, MO, USA) for 15 minutes at room temperature in the dark. Slides were mounted with a non fluorescent mounting medium Cytifluor (Cytifluor, London, UK). A negative control experiment was performed by omitting the rTdT enzyme in the incubation buffer.

### Cell proliferation assay

To assess the ability of cells to proliferate in MG and *in vitro* the 5-Bromo-2′-deoxy-uridine we used Labelling and Detection Kit I (Roche, Basel, Switzerland). Immediately after dissection, MG pellets were incubated with 5-Bromo-2′-deoxy-uridine (BrdU) 10 µM in cell culture medium for 1 hour at 20 °C. After PBS washes, MG implants were embedded in a tissue freezing medium (OCT, Tebu-Bio, Italy) and immediately frozen in liquid nitrogen and sectioned with a cryotome (Leica CM 1850, Wetzlar, Germany). *In vitro* treated macrophages were incubated in the same conditions immediately after treatment and then fixed with paraformaldehyde 4% for 15 minutes at room temperature.

For the immunofluorescence procedure, specimens were incubated with Anti-BrdU working solution (Roche, Basel, Switzerland) for 30 minutes at 37 °C and then washed with the provided washing buffer. Afterwards an anti-mouse DyLight 549 conjugated antibody (KPL, Gaithersburg, MD, USA) was used for MG slides while an anti-mouse-Ig-fluorescein antibody (Roche, Basel, Switzerland) was used for macrophages. Specimens were incubated with the secondary antibody for 30 minutes at 37 °C in the dark. After further washes, nuclei were counterstained with 4′,6-diamidino-2-phenylindole (DAPI) (Sigma Aldrich, Saint Luis, MO, USA) for 5 minutes at room temperature in the dark.

Slides were mounted with Cytifluor (Cytifluor, London, UK).

### Intracellular ROS evaluation

ROS production was evaluated after *in vitro* treatment using 2′,7′-dichlorodihydrofluorescein diacetate (H_2_DCFDA) (Molecular Probes, Eugene, OR, USA), a fluorigenic probe commonly used to detect intracellular ROS. H_2_DCFDA is a non-fluorescent compound, able to cross cell membranes. Once within the cell it is hydrolyzed to 2′,7′-dichlorofluorescein (DCF), a compound that becomes fluorescent when it is oxidized by ROS. ROS level can be detected by monitoring the increase in fluorescence. Treated and non-treated macrophages were washed with Hank’s balanced salt (HBSS) solution (Sigma Aldrich, Saint Luis, MO, USA) and then incubated with 10 µM H_2_DCFDA for 30 min at 20 °C in the dark. Cells were then washed 3 times in HBSS and fixed with paraformaldehyde 4% for 15 minutes. Nuclei were stained with DAPI.

### Images acquisition and recording

Slides were observed under a light/fluorescence microscope Nikon Eclipse Ni (Nikon, Tokyo, Japan) equipped with three different excitation/emission filters: 360/420 nm, for DAPI nuclear staining; 488/525 nm, for H_2_DCFDA, and fluorescein stainings; 550/580 nm, for propidium iodide, Cy3 and DyLight 549 signals.

Data were recorded with Nikon Digital Sight DS-DM digital camera (Nikon, Tokyo, Japan) and images were combined with Adobe Photoshop (Adobe Systems, San Jose, CA, USA).

### Statistical analysis

The percentages of positive and negative cells after BrdU and TUNEL assays, were assessed by analysing 3 different slides (10 random fields of 45000 μm^2^ for each slide) using the Image J software package. Nuclei in each field were counted and classified as positive or negative. The percentage of positive nuclei on the total count was then calculated. Data represent the mean SEM of the total percentages. The fluorescence intensity of H_2_DCFDA in ROS assay was measured by analysing 3 different slides for each time point/assay (five random fields for each slide) using the Image J software package.

Statistical analysis were performed using Statistica 7.0 software (StatSoft, Inc., Tulsa, OK, USA). Statistical differences were calculated by Factorial ANOVA followed by Tukey’s post-hoc test and p < 0.05 was considered statistically significant.

### Data availability

All data generated or analysed during this study are included in this published article.
